# Endo-DET: A Domain-Specific Detection Framework for Multi-Class Endoscopic Disease Detection

**DOI:** 10.3390/jimaging12030112

**Published:** 2026-03-06

**Authors:** Yijie Lu, Yixiang Zhao, Qiang Yu, Wei Shao, Renbin Shen

**Affiliations:** 1 The Second School of Clinical Medicine, Nanjing Medical University, Nanjing 211166, China; luyijie@stu.njmu.edu.cn; 2 School of Public Health, Nanjing Medical University, Nanjing 211166, China; zhaoyixiang@stu.njmu.edu.cn; 3 Department of Gastroenterology, The Affiliated Suzhou Hospital of Nanjing Medical University, Suzhou Municipal Hospital, Suzhou 215002, China; 4 The Affiliated Suzhou Hospital of Nanjing Medical University, Suzhou Municipal Hospital, Gusu School, Nanjing Medical University, Suzhou 215001, China; 5 Department of Gastrointestinal Surgery, The Affiliated Suzhou Hospital of Nanjing Medical University, Suzhou Municipal Hospital, Suzhou 215002, China

**Keywords:** endoscopic disease detection, deep learning, Transformer, DEIM, illumination correction, multi-class detection, gastrointestinal cancer, computer-aided diagnosis

## Abstract

Gastrointestinal cancers account for roughly a quarter of global cancer incidence, and early detection through endoscopy has proven effective in reducing mortality. Multi-class endoscopic disease detection, however, faces three persistent challenges: feature redundancy from non-pathological content, severe illumination inconsistency across imaging modalities, and extreme scale variability with blurry boundaries. This paper introduces Endo-DET, a domain-specific detection framework addressing these challenges through three synergistic components. The Adaptive Lesion-Discriminative Filtering (ALDF) module achieves lesion-focused attention via sparse simplex projection, reducing complexity from $O(N^{2})$ to $O(\alpha N^{2})$. The Global–Local Illumination Modulation Neck (GLIM-Neck) enables illumination-aware multi-scale fusion through four cooperative mechanisms, maintaining stable performance across white-light endoscopy, narrow-band imaging, and chromoendoscopy. The Lesion-aware Unified Calibration and Illumination-robust Discrimination (LUCID) module uses dual-stream reciprocal modulation to integrate boundary-sensitive textures with global semantics while suppressing instrument artifacts. Experiments on EDD2020, Kvasir-SEG, PolypGen2021, and CVC-ClinicDB show that Endo-DET improves mAP50-95 over the DEIM baseline by 5.8, 10.8, 4.1, and 10.1 percentage points respectively, with mAP75 gains of 6.1, 10.3, 6.8, and 9.3 points, and Recall50-95 improvements of 10.9, 12.1, 11.1, and 11.5 points. Running at 330 FPS with TensorRT FP16 optimization, Endo-DET achieves consistent cross-dataset improvements while maintaining real-time capability, providing a methodological foundation for clinical computer-aided diagnosis.

## 1. Introduction

Gastrointestinal cancers account for about a quarter of global cancer cases and about a third of cancer deaths [1]. Early detection via endoscopic monitoring has shown promise in reducing colorectal cancer-related mortality, highlighting the prognostic benefit of identifying disease at an earlier stage [2]. Clinical miss rates remain troublingly high: colorectal polyp miss rates range from 6% to 27%, and gastric cancer miss rates exceed 9.2% [3,4]. These miss rates stem from physician experience variability, visual fatigue during prolonged examinations, and the morphological diversity of lesions. Computer-aided detection systems offer an objective and quantitative technical solution [5].

Deep learning has made significant strides in gastrointestinal disease detection. Misawa et al. [6] developed a polyp detection system based on YOLOv3 achieving over 90% sensitivity and specificity on 56,668 training images. Karaman et al. [7] improved detection accuracy on the SUN and PICCOLO datasets by optimising YOLO parameters. Zhang et al. [8] proposed an improved Mask R-CNN for early gastric cancer detection. He et al. [9] developed the ENDOANGEL-ME system with 90.32% diagnostic accuracy, outperforming senior physicians at 70.16%. Ali et al. [10,11] used the EndoCV2020 and PolypGen challenges to reveal core issues, including class imbalance, scale variation, and multi-modal differences.

However, existing research hits three fundamental walls when it comes to clinical applications. Single-class methods generalize poorly and tend to misclassify lesions when multiple disease categories coexist [10]. Cross-modal robustness also remains insufficient. Chromatic shifts across white-light endoscopy (WLE), narrow band imaging (NBI), and chromoendoscopy (CE) contaminate feature representations [12]. Dataset quality and scale are limiting factors. The field lacks high-quality, multi-class, cross-modal large-scale datasets. Existing datasets like EDD2020 and Kvasir-SEG have limited scale and severe class imbalance. Hyper-Kvasir offers larger scale but lacks high-quality annotations. This constraint represents perhaps the key obstacle preventing endoscopic AI from lab to clinic.

Recent Transformer-based detectors have advanced the state of the art in natural image benchmarks. DINO [13] introduces contrastive denoising training and mixed query selection, achieving strong performance on COCO. Co-DETR [14] further improves encoder learning through collaborative hybrid assignments. D-FINE [15] redefines bounding box regression as fine-grained distribution refinement, and DEIM [16] addresses sparse supervision through dense O2O matching. While these methods push accuracy–speed trade-offs on general benchmarks, their architectures remain agnostic to the domain-specific challenges of endoscopic imaging: none of them incorporate mechanisms for cross-modal illumination normalization, lesion-focused sparse attention, or artifact-aware feature discrimination. Directly applying these general-purpose detectors to multi-class endoscopic disease detection yields suboptimal results, as confirmed by our experiments, motivating the domain-specific design of Endo-DET.

Current gastrointestinal disease diagnosis algorithms fall into three main categories. Instance segmentation methods [17] provide precise boundaries but require 10–15 times the annotation cost of bounding boxes. They have heavy computational overhead, and medical segmentation datasets are even scarcer. Semantic segmentation methods [18] cannot distinguish between instances of the same class and tend to misclassify small lesions as background under class imbalance. Object detection methods [6,7] offer moderate data requirements, fast inference, and the ability to distinguish multiple instances, making them better suited for real-time clinical scenarios. We therefore adopt the object detection paradigm for real-time multi-class endoscopic disease detection.

Despite these advantages, object detection still faces domain-specific challenges in multi-class endoscopic disease detection. Lesions span a wide range of scales, from sub-millimeter dysplastic patches to centimeter-scale polyps, yet occupy only small portions of the visual field. Standard detection architectures waste considerable computation on non-diagnostic regions. Cross-modal illumination inconsistency is severe: chromatic differences, specular reflections, and shadows across WLE, NBI, and CE cause substantial intensity variations for the same lesion. Standard Feature Pyramid Network (FPN) style neck networks cannot achieve stable cross-modal representation. Morphologically similar lesion categories are difficult to distinguish. Barrett’s esophagus, suspicious lesions, and high-grade dysplasia exist on a continuum, and blurry boundaries plus instrument artifacts make feature discrimination even harder.

To address these challenges, we propose Endo-DET, a domain-specific framework for multi-class endoscopic disease detection. The framework does not simply pursue maximum detection accuracy. Instead, it systematically addresses the problems in the core technical detail by architectural innovation and establishing the methodological foundation for clinical deployment. The core idea is as follows: on limited-scale multi-class datasets, precise adaptation to endoscopic scene features is performed by domain-specific modules rather than capacity expansion.

Endo-DET provides solutions through three synergistic components. Adaptive Lesion-Discriminative Filtering (ALDF) allows for lesion-focused sparse attention. Global–Local Illumination Modulation Neck (GLIM-Neck) delivers cross-modal illumination-aware fusion. Lesion-aware Unified Calibration and Illumination-robust Discrimination (LUCID) offers fine-grained feature refinement.

Our specific contributions are as follows:

(1) We design Endo-DET, a DEIM based multiclass endoscopic disease detection architecture addresses feature redundancy, illumination inconsistency and feature confusion. On an EDD2020 benchmark, it achieves a 5.8% performance improvement over the baseline and demonstrates generalization across three independent datasets: Kvasir-SEG, PolypGen2021, and CVC-ClinicDB.
(2) To mitigate illumination inconsistency on imaging modalities, we design GLIM-Neck. This module achieves illumination-aware multi-scale feature fusion through four cooperative mechanisms, keeping detection performance stable across WLE, NBI and CE.
(3) To handle blurry boundaries due to morphologically similar lesion classes, we design the LUCID module based on dual-stream reciprocal modulation. It combines boundary-sensitive local textures with discriminative global semantics while suppressing spurious responses from non-diagnostic patterns like instrument artifacts.
(4) We perform comprehensive experiments on four datasets, demonstrating that Endo-DET achieves consistent cross-dataset performance improvements while maintaining real-time inference capability at 330 FPS. This confirms domain specific design and reveals how current dataset scale and quality fundamentally limit performance ceilings.

## 2. Related Work

With advances in deep learning, the automatic detection of gastrointestinal diseases has exploded significantly. In this section we review detection methods from Two-Stage- to Transformer-based architectures, considering their applications and limitations in endoscopic scenarios. We then examine the domain-specific enhancement techniques which directly inspired the core components of Endo-DET.

### 2.1. Evolution of Object Detection Methods

Object detection methods divide into three major paradigms. Two-Stage detectors, represented by Faster R-CNN [19], generate candidate boxes through region proposal networks before classification and regression. They are widely used in medical image analysis [8] but have computational overhead, making real-time clinical deployment difficult.

One-Stage detectors like the YOLO series [20,21,22] achieve end-to-end detection and have excellent speed–accuracy trade-offs in polyp detection tasks [6,7]. However, their one-to-many label assignment process produces redundant predictions requiring complex post-processing.

DEtection TRansformer [23] introduced a novel end-to-end detection paradigm using Hungarian matching for one-to-one label assignment, eliminating dependence on non-maximum suppression. Deformable-DETR [24] resolved the $O(N^{2})$ complexity of standard attention through deformable cross-attention, accelerating convergence. DINO [13] further advanced DETR-series performance through contrastive denoising training, mixed query selection, and look-forward-twice strategies, establishing a new state of the art for COCO. Co-DETR [14] improved encoder feature learning by introducing collaborative hybrid assignments from multiple parallel auxiliary heads, demonstrating that versatile label assignment can enhance one-to-one matching. RT-DETR [25] achieved real-time DETR performance through a hybrid encoder decoupling intra-scale and cross-scale interactions. RT-DETRv2 [26] extended this with flexible decoder tuning and improved training strategies. D-FINE [15] redefined bounding box regression as fine-grained distribution refinement, achieving strong accuracy–speed trade-offs. These advances collectively pushed Transformer-based detection toward real-time deployment, providing the architectural foundation upon which DEIM [16] and our Endo-DET are built. However, all these methods target general object detection and lack domain-specific adaptation for the unique challenges of endoscopic imaging.

### 2.2. Our Baseline Model: DEIM

DEIM [16] is a recently proposed, efficient real-time detection framework that provides the architectural foundation for Endo-DET. DEIM addresses the sparse supervision problem in DETR-series models’ one-to-one (O2O) matching through two core innovations.

Dense O2O Matching Strategy: In traditional O2O matching, only a few queries per image match the ground truth targets, leading to sparse supervision and slow convergence. DEIM introduces additional targets by standard data augmentation. It increases positive samples per image and accelerates model convergence. This characteristic is particularly useful for endoscopic disease detection. The EDD2020 dataset contains only 385 images with 749 annotated instances, making the sparse supervision problem even more acute. Dense O2O extends effective training samples by augmentation strategies, easing training difficulties on small-scale medical datasets.

Matchability-Aware Loss (MAL): Dense O2O matching speeds convergence but introduces many low-quality matches which may affect detection performance. MAL adaptively optimizes matches at different quality levels to improve Dense O2O performance. This is important for the blurry boundary lesions in endoscopic images, illumination-disturbed regions, and morphologically similar classes. The model maintains the high recall and precise localization.

Performance and Efficiency Balance: On the COCO dataset, DEIM combined with RT-DETR and D-FINE [15] reduces training time by 50% while continuously improving performance. DEIM-D-FINE-L achieves 54.7% AP at 124 FPS on an NVIDIA T4 GPU, while DEIM-D-FINE-X reaches 56.5% AP at 78 FPS. This excellent speed–accuracy trade-off shows its feasibility for real-time clinical deployment.

We chose DEIM-nano for several reasons. Its Dense O2O approach fits the sparse positive sample problem in small medical datasets. The MAL loss handles the heterogeneous matching quality, matching the complexity of endoscopic images. Its lightweight architecture meets real-time analysis requirements. Its modular design facilitates the integration of domain-specific ALDF, GLIM-Neck, and LUCID components.

## 3. Methods

### 3.1. Overall Architecture

Multi-class endoscopic disease detection in real clinical applications faces three key challenges. Large amounts of non-pathological content cause feature redundancy. Normal mucosal tissue, anatomical structures, and imaging artifacts dominate the visual field, while diagnostically relevant lesion regions are sparsely distributed. Severe illumination inconsistency exists across imaging modalities. White-light endoscopy (WLE), narrow band imaging (NBI) and chromoendoscopy (CE) make identical lesions appear very different visually. Chromatic shifts, intensity variations, and specular reflections on mucosal surfaces alter feature representations. Extreme scale variability with blurry boundaries runs through disease stages, from sub-millimeter early dysplastic patches to centimeter-scale late stage lesions, which have unclear boundaries that challenge standard detection architectures.

Compliant computer vision techniques have proposed separate solutions for these challenges: sparse attention schemes for computational efficiency, multi-scale feature pyramids for scale variation, and dual-stream architectures for feature discrimination. However, their direct application to endoscopic disease detection encounters domain specific challenges. Cross-modal endoscopic datasets have constraints such as limited annotation density relative to natural images, extreme class imbalance with severe under-representation of rare but clinically critical classes like early cancer and high-grade dysplasia, and multi-modal imaging protocols that introduce systematic appearance variations beyond natural scene illumination changes.

To address these challenges through domain-specific adaptation, we designed Endo-DET, integrating three synergistic components. Adaptive Lesion-Discriminative Filtering (ALDF) achieves lesion-focused token selection through sparse simplex projection. Global–Local Illumination Modulation Neck (GLIM-Neck) enables illumination-aware multi-scale fusion through four cooperative mechanisms. Lesion-aware Unified Calibration and Illumination-robust Discrimination (LUCID) achieves feature refinement through reciprocal gating between detail and context streams.

**Figure 1:**
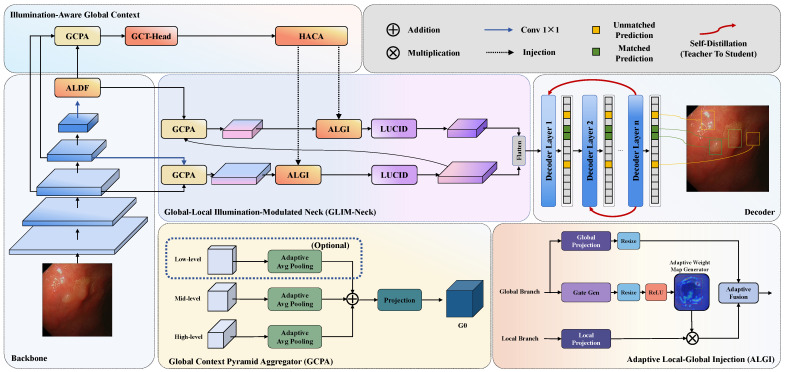
Overall architecture of Endo-DET. The framework comprises four main components: backbone network for hierarchical feature extraction, ALDF-enhanced Transformer encoder for lesion-focused sparse attention, GLIM-Neck for illumination-aware multi-scale fusion, and LUCID for dual-stream feature refinement.

The overall framework appears in Figure 1. Given an endoscopic image of size H×W×3, the architecture includes four main components. The backbone extracts hierarchical features $\left\{F_{8},F_{16},F_{32}\right\}$ at different resolutions. The ALDF-enhanced Transformer encoder provides lesion-focused sparse attention with lower computational cost. GLIM-Neck integrates illumination-aware global context through pyramid aggregation, Transformer refinement, hierarchical channel allocation, and adaptive local–global injection. LUCID calibrates features using dual-stream reciprocal modulation.

The framework builds on the DEIM architecture, leveraging dense O2O matching for the sparse supervision problems inherent to medical image datasets. We detail the design rationale and mathematical formulation of each component below.

### 3.2. Adaptive Lesion-Discriminative Filtering Module (ALDF)

Standard Transformer encoders in detection architectures employ dense global self-attention with $O(N^{2})$ computational complexity, treating all spatial token positions uniformly. Endoscopic disease detection, however, presents fundamentally different spatial distributions compared to natural object detection. Diagnostically relevant lesion regions occupy sparse, localized areas while non-pathological content, including normal mucosa, anatomical landmarks, imaging artifacts, and surgical instruments, dominates most spatial positions. Dense attention mechanisms allocate substantial computation to background regions lacking diagnostic information, diluting feature saliency from sparse lesion patterns and failing to prioritize the token interactions that are most relevant to medical diagnosis.

To address this spatial redundancy while maintaining detection sensitivity, we adapt sparse attention through grouped semantic modeling and learnable simplex projection, enabling a selective focus on lesion-discriminative token subsets.

**Figure 2:**
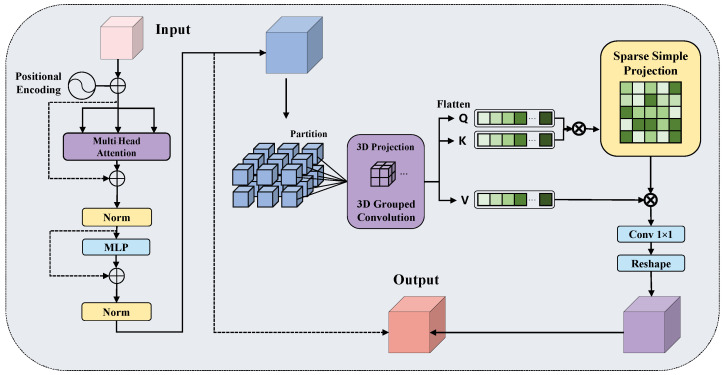
Architecture of Adaptive Lesion-Discriminative Filtering (ALDF) module. The module performs grouped channel decomposition, hybrid convolutional projection for Q/K/V generation, and learnable sparse simplex projection for adaptive top-k token filtering.

ALDF integrates three cooperative mechanisms: grouped channel decomposition for simultaneous multi-scale semantic modeling, hybrid convolutional projection for local spatial context injection, and learnable sparse simplex projection for adaptive top-k token filtering. The detailed architecture appears in Figure 2, showing the complete computational flow from input feature transformation through attention score computation to sparse token aggregation.

Given input features $F\in R^{B\times C\times H\times W}$ augmented with learnable positional encoding, we decompose the channel dimension into *G* semantic groups to enable the parallel processing of features at different semantic abstraction levels:

(1) $$F_{g}=\Psi_{\mathrm{reshape}}\left(F\right)\in R^{B\times G\times (C/G)\times H\times W}$$

where $\Psi_{\mathrm{reshape}}\left(\cdot \right)$ implements channel-to-group transformation, with each group capturing feature patterns at different semantic scales. This grouped decomposition facilitates cross-scale token interaction within a unified attention space while reducing per-group computational cost.

Query, Key, and Value projections generate through a hybrid convolutional architecture combining 3D grouped convolution $\Phi_{3D}\left(\cdot \right)$ with depthwise separable convolution $\Phi_{\mathrm{DW}}\left(\cdot \right)$:

(2) $$Q,K,V=\Psi_{\mathrm{split}}\left(\Phi_{\mathrm{DW}}\left(\Phi_{3D}\left(F_{g}\right)\right),3\right)$$

where $\Phi_{3D}\left(\cdot \right)$ operates on grouped channels with 1×3×3 kernels, $\Phi_{\mathrm{DW}}\left(\cdot \right)$ applies depthwise separable convolution for local context aggregation, and $\Psi_{\mathrm{split}}\left(\cdot ,3\right)$ splits output into three equal components. Grouped features then flatten into token sequences of length N=H×W×G forming $T\in R^{B\times N\times D}$, where each token simultaneously encodes spatial position and semantic group membership.

To stabilize attention computation and prevent gradient saturation, we apply $\ell_{2}$ normalization to Query and Key representations with learnable temperature modulation:

(3) $$\hat{Q}=Q/{∥Q∥}_{2},\;\hat{K}=K/{∥K∥}_{2}$$

(4) $$S=\left({\hat{Q}}^{⊤}\hat{K}\right)⊙\tau \in R^{N\times N}$$

where ${∥\cdot ∥}_{2}$ denotes $\ell_{2}$ norm, ⊙ denotes element-wise multiplication, and $\tau \in R^{+}$ is a learnable temperature parameter initialized at $\tau_{0}=\sqrt{D}$. Normalized attention scores *S* quantify semantic affinity between all token pairs while maintaining numerical stability through bounded magnitudes.

To achieve computational efficiency without sacrificing detection sensitivity, we introduce learnable sparse simplex projection. For each query token $q_{i}$, we construct sparse attention patterns by retaining only top-*k* key tokens exhibiting highest semantic affinity. Given N=H×W×G tokens, the number of retained tokens is

(5) k=⌊α·N⌋

where α is the retention ratio that directly controls the computational complexity ratio between sparse and dense attention:

(6) $$\frac{{\mathrm{FLOPs}}_{\mathrm{sparse}}}{{\mathrm{FLOPs}}_{\mathrm{dense}}}=\frac{\alpha N^{2}D}{N^{2}D}=\alpha$$

We set α=0.8 in all experiments, yielding a 20% reduction in attention computation. This relatively conservative pruning reflects a domain-specific consideration: in endoscopic images, lesion regions are sparsely distributed but their spatial extent varies substantially, from sub-millimeter dysplastic patches to centimeter-scale polyps. Aggressive pruning (α≤0.5) risks discarding tokens at lesion peripheries where boundary-discriminative information resides, directly harming localization precision. Conversely, α→1.0 approaches dense attention with a negligible efficiency gain. At α=0.8, the top-*k* selection provides sufficient spatial coverage to capture the total extent of the lesion, including boundary regions, consistent with the clinical priority of minimizing false negatives during medical screenings. This is formalized through a binary selection mask:

(7) $$M\in {\left\{0,1\right\}}^{N\times N},\;M_{ij}=⊮\left\{j\in \Psi_{\mathrm{TopK}}\left(S_{i,:},k\right)\right\}$$

where $\Psi_{\mathrm{TopK}}\left(\cdot ,k\right)$ selects indices of *k* largest elements and ⊮{·} is the indicator function. Sparse attention weights compute through masked softmax normalization:

(8) $$A=\Psi_{\mathrm{softmax}}\left(S⊙M+\left(1-M\right)⊙\left(-\infty \cdot 1\right)\right)$$

where unselected positions map to negative infinity, ensuring zero contribution in softmax operation, ⊙ denotes element-wise multiplication, and 1 is an all-ones matrix. This formalization achieves hard attention selection while maintaining differentiability through softmax operation.

Output features are obtained through attention-weighted value aggregation, projection, and residual connection:

(9) $$Z=AV^{⊤}\in R^{N\times D}$$

(10) $$F_{\mathrm{out}}=F+\Phi_{\mathrm{proj}}\left(\Psi_{\mathrm{reshape}}\left(Z\right)\right)$$

where $\Phi_{\mathrm{proj}}\left(\cdot \right)$ denotes 1×1 projection convolution mapping aggregated features back to original spatial configuration, and $\Psi_{\mathrm{reshape}}\left(\cdot \right)$ converts the token sequence to spatial feature map format.

As a core encoder component, ALDF improves lesion discrimination by learning sparse patterns while suppressing computational redundancy in background regions. Sparse simplex projection reduces attention complexity from $O(N^{2}\cdot D)$ to $O(\alpha \cdot N^{2}\cdot D)$, achieving significant efficiency gains when α<0.7. Grouped channel decomposition provides additional computational savings, with FLOPs proportional to $H\times W\times k^{2}\times C^{2}/G$, where *k* denotes kernel size and *G* denotes group count.

Detection sensitivity is a priority over computational pruning, since medical screening applications need high recall to minimize false negatives in rare disease detection. Learnable temperature parameter τ and retention ratio α allow for adaptive attention pattern formation according to endoscopic lesion characteristics. ALDF distinguishes generic sparse attention for natural image understanding.

### 3.3. Global–Local Illumination Modulation Neck (GLIM-Neck)

Cross-modal endoscopic imaging protocols introduce severe illumination inconsistency that fundamentally challenges standard multi-scale feature fusion architectures. White-light endoscopy captures broadband visible spectrum information. Narrow-band imaging selectively filters wavelengths to enhance vascular patterns. Chromoendoscopy applies topical dyes that significantly alter tissue chromaticity. Standard feature pyramid networks propagate entangled illumination-structure representations across scales, where specular reflections on moist mucosal surfaces, shadows from camera position changes, and directional anatomical folds create spatially heterogeneous illumination distributions contaminating hierarchical features.

Existing illumination normalization methods apply spatially uniform correction, risking either over-processing diagnostically relevant color variations at lesion boundaries, where subtle chromaticity encodes tissue pathology, or under-processing background artifacts where strong reflections persist. To address this limitation through content-adaptive processing, we designed GLIM-Neck to implement four cooperative mechanisms for illumination-aware hierarchical feature integration.

GLIM-Neck comprises four cascaded components. The Global Context Pyramid Aggregator (GCPA) constructs lesion-aware multi-scale context through adaptive pooling and feature concatenation. Global Context Transformer Head (GCT-Head) refines aggregated context through self-attention to learn illumination-invariant representations. Hierarchical Asymmetric Channel Allocation (HACA) decomposes refined context into pyramid level-specific guidance with differentiated channel capacity. Adaptive Local-Global Injection (ALGI) enables learnable data-driven fusion between local structural features and global illumination context.

**Figure 3:**
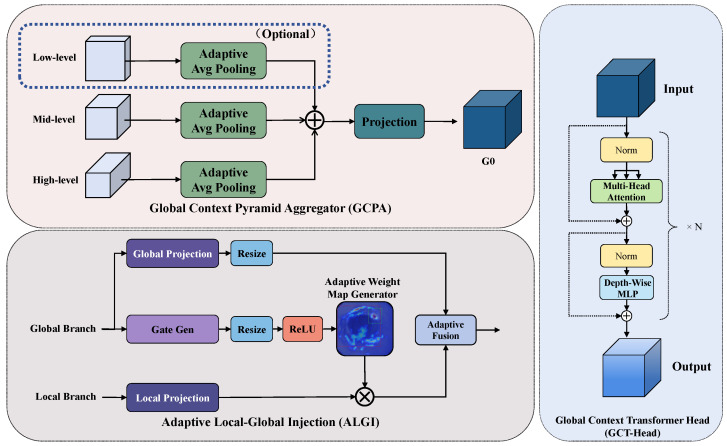
Internal component design of GLIM-Neck. **Top Left**: GCPA unifies multi-resolution features through adaptive pooling and 1×1 projection. **Right**: GCT-Head refines global context through L=2 Transformer blocks with multi-head attention and depth-wise MLP. **Bottom Left**: ALGI performs adaptive local–global fusion through parallel branches with a learned fusion coefficient λ. See Figure 4 for the overall data flow.

**Figure 4:**
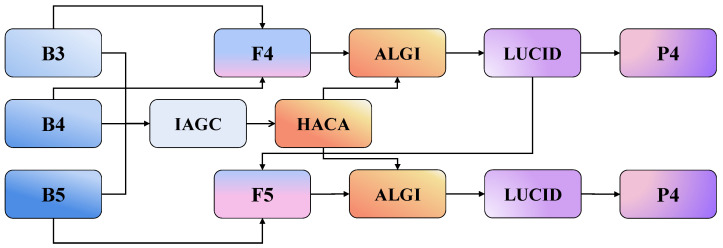
Data flow of GLIM-Neck. Backbone outputs B3 (stride 8) and B4 (stride 16) together with ALDF-enhanced feature B5 (stride 32) enter the illumination-aware global context (IAGC) branch. HACA splits the refined context into level-specific guidance, which ALGI injects back into each pyramid level. LUCID modules subsequently refine P4 and P5 before feeding into the decoder.

The complete architecture appears in Figure 3 and Figure 4, depicting information flow from multi-resolution feature aggregation through Transformer refinement to adaptive hierarchical injection.

For the main processing branch receiving multi-resolution features $F_{\mathrm{fine}}\in R^{C_{1}\times H_{1}\times W_{1}}$, $F_{\mathrm{mid}}\in R^{C_{2}\times H_{2}\times W_{2}}$, and $F_{\mathrm{ALDF}}\in R^{C_{3}\times H_{3}\times W_{3}}$ from fine (stride 8), middle (stride 16), and coarse (stride 32) levels respectively, GCPA performs resolution unification through adaptive pooling followed by channel-wise aggregation:

(11) $$G_{0}=\Phi_{1\times 1}\left(\Psi_{\mathrm{concat}}\left(\Psi_{\mathrm{pool}}\left(F_{\mathrm{fine}},H_{c}\right),\Psi_{\mathrm{pool}}\left(F_{\mathrm{mid}},H_{c}\right),\Psi_{\mathrm{pool}}\left(F_{\mathrm{ALDF}},H_{c}\right)\right)\right)\in R^{C_{g}\times H_{c}\times W_{c}}$$

where $\Phi_{1\times 1}\left(\cdot \right)$ implements 1×1 projection convolution, $\Psi_{\mathrm{pool}}\left(\cdot ,H_{c}\right)$ denotes adaptive average pooling to target resolution $H_{c}\times W_{c}$, $\Psi_{\mathrm{concat}}\left(\cdot \right)$ executes channel-wise concatenation, and $C_{g}$ denotes the projected channel dimension. The integration of $F_{\mathrm{ALDF}}$, the lesion-discriminative filtered features, ensures the aggregated global context $G_{0}$ encodes both global illumination statistics and spatial priors regarding diagnostically salient regions, extending traditional pyramid fusion with lesion awareness.

GCT-Head refines aggregated global context $G_{0}$ through a lightweight Transformer encoder comprising L=2 stacked blocks with 4-head self-attention and feed-forward hidden dimension $4C_{g}$. Each block integrates multi-head self-attention with depth-enhanced feed-forward networks:

(12) $$G^{\left(\ell \right)}=G^{\left(\ell -1\right)}+\Psi_{\mathrm{MHSA}}\left(\Phi_{\mathrm{LN}}\left(G^{\left(\ell -1\right)}\right)\right)$$

(13) $$G^{\left(\ell \right)}=G^{\left(\ell \right)}+\Psi_{\mathrm{DW}-\mathrm{FFN}}\left(\Phi_{\mathrm{LN}}\left(G^{\left(\ell \right)}\right)\right)$$

where $\Phi_{\mathrm{LN}}\left(\cdot \right)$ denotes layer normalization, $\Psi_{\mathrm{MHSA}}\left(\cdot \right)$ implements multi-head self-attention enabling global information aggregation, $\Psi_{\mathrm{DW}-\mathrm{FFN}}\left(\cdot \right)$ denotes depth-enhanced feed-forward network introducing local inductive bias, and ℓ∈{1,2,…,L} indexes layers.

Refined global context $G=G^{\left(L\right)}$ effectively captures spatially correlated patterns, where neighboring regions typically share similar illumination while specular highlights and cast shadows manifest as localized discontinuities. Since $G_{0}$ integrates ALDF-enhanced features encoding lesion spatial priors, refined representation *G* distinguishes intensity variations attributable to illumination artifacts from tissue reflectance differences attributable to pathological changes.

HACA implements pyramid-level-specific channel capacity allocation based on the observation that different resolutions serve different detection objectives. High-resolution levels (stride 8) handle precise boundary localization and small-lesion detection, requiring local detail preservation. Low-resolution levels (stride 32) support large-lesion detection and global semantic understanding, requiring comprehensive contextual information.

HACA decomposes refined global context *G* through learned projection and channel-wise splitting:

(14) $$\left[G^{\left(1\right)};G^{\left(2\right)};\ldots ;G^{\left(K\right)}\right]=\Psi_{\mathrm{split}}\left(\Phi_{\mathrm{HACA}}\left(G\right),\left[C_{1},C_{2},\ldots ,C_{K}\right]\right)$$

where *K* denotes pyramid level count and $C_{k}$ denotes channel allocation for level *k*. In this work, K=2 corresponding to stride-16 and stride-32 feature levels, with linear scaling allocation:

(15) $$C_{k}=k\cdot C_{\mathrm{base}},\;C_{\mathrm{base}}=\frac{C_{g}}{\sum_{k=1}^{K}k}=\frac{C_{g}}{3}$$

yielding $C_{1}=C_{g}/3$ and $C_{2}=2C_{g}/3$. This asymmetric allocation reflects the information density asymmetry across pyramid levels. The stride-32 level encodes global semantic context over large receptive fields, where illumination trends, lesion category semantics, and inter-class discrimination require richer channel capacity. The stride-16 level primarily relays spatially precise boundary information, which is inherently lower-dimensional and can be adequately represented with fewer channels. The linear scaling rule $C_{k}\propto k$ provides a simple heuristic: each successive pyramid level receives proportionally more channels, avoiding both the under-parameterization of semantically rich coarse levels and over-parameterization of spatially precise fine levels. $\Phi_{\mathrm{HACA}}\left(\cdot \right)$ implements learned channel projection, and $\Psi_{\mathrm{split}}\left(\cdot ,\cdot \right)$ splits along the channel dimension according to the above ratios.

ALGI enables content-adaptive local–global integration through learned fusion coefficients at each pyramid level *ℓ*. Given local features $L_{\ell }$ and pyramid-specific guidance $G^{\left(\ell \right)}$ from HACA, dual-branch architecture processes local and global information in parallel:

(16) $$f_{\ell }^{\mathrm{local}}=\Phi_{\mathrm{local}}\left(L_{\ell }\right)$$

(17) $$a_{\ell }=\sigma_{h}\left(\Psi_{\mathrm{resize}}\left(\Phi_{\mathrm{act}}\left(G^{\left(\ell \right)}\right)\right)\right),\;f_{\ell }^{\mathrm{global}}=\Psi_{\mathrm{resize}}\left(\Phi_{\mathrm{global}}\left(G^{\left(\ell \right)}\right)\right)$$

where $\Phi_{\mathrm{local}}\left(\cdot \right)$, $\Phi_{\mathrm{act}}\left(\cdot \right)$, and $\Phi_{\mathrm{global}}\left(\cdot \right)$ denote 1×1 projection convolutions, $\Psi_{\mathrm{resize}}\left(\cdot \right)$ achieves resolution alignment through adaptive pooling or bilinear interpolation, and $\sigma_{h}\left(\cdot \right)$ denotes hard sigmoid activation.

Adaptive fusion mechanism enables dynamic balance between local and global branches through learned parameters. Learnable parameter $\theta_{\ell }\in R$ controls fusion coefficient $\lambda_{\ell }$ through sigmoid mapping:

(18) $$\lambda_{\ell }=\sigma \left(\theta_{\ell }\right)\in \left(0,1\right)$$

supporting continuous interpolation between branch contributions. The final output achieves soft switching through convex combination:

(19) $$Y_{\ell }=F_{\mathrm{adaptive}}\left(f_{\ell }^{\mathrm{local}},f_{\ell }^{\mathrm{global}},a_{\ell };\theta_{\ell }\right)=\lambda_{\ell }\cdot \left(f_{\ell }^{\mathrm{local}}⊙a_{\ell }\right)+\left(1-\lambda_{\ell }\right)\cdot f_{\ell }^{\mathrm{global}}$$

where ⊙ denotes element-wise multiplication.

This formalization exhibits clear interpretability. When $\lambda_{\ell }\to 1$, fusion emphasizes gated local features, preserving boundary details and fine textures, making it suitable for regions with stable illumination and strong local discriminability. When $\lambda_{\ell }\to 0$, fusion favors the global context, achieving stronger illumination normalization, making it suitable for regions with severe illumination artifacts requiring global semantic compensation. Parameter $\theta_{\ell }$ undergoes end-to-end optimization, enabling the network to discover task-optimal fusion strategies adapted to specific pyramid levels and input content characteristics, achieving a dynamic balance between detail fidelity and illumination robustness.

### 3.4. Lesion-Aware Unified Calibration and Illumination-Robust Discrimination Module (LUCID)

After lesion-focused encoding and illumination-aware hierarchical fusion, feature representations may still contain spurious activations from non-diagnostic patterns including surgical instruments like biopsy forceps and suction catheters, imaging artifacts like bubbles, mucus deposits, and fluid residue, specular reflections on moist mucosal surfaces, and repetitive anatomical structures like mucosal folds and vascular patterns. These interfering patterns exhibit local textural similarity to pathological lesions while differing fundamentally in semantic context and spatial distribution characteristics.

Existing single-path feature refinement architectures struggle to simultaneously capture boundary-sensitive local details, which are crucial for precise localization, and discriminative global semantic context, which is crucial for distinguishing genuine lesions from artifacts. To address this through complementary processing pathways, we designed LUCID to implement dual-stream reciprocal modulation with progressive channel refinement for unified lesion-aware calibration.

LUCID comprises three sequentially operating mechanisms. Channel-wise stream splitting separates input features into specialized detail and context processing paths. Reciprocal gating enables bidirectional cross-stream modulation, where context generates spatial attention guiding detail stream’s spatial focus while detail generates channel attention guiding the context stream’s channel selection. Progressive channel sculpting further refines representations through spatial gating units, suppressing non-diagnostic response patterns.

**Figure 5:**
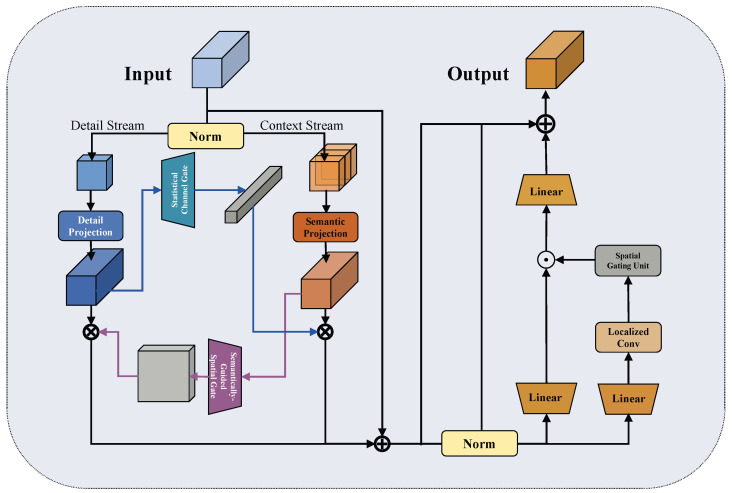
Architecture of the Lesion-aware Unified Calibration and Illumination-robust Discrimination (LUCID) module. The module performs channel-wise stream splitting, reciprocal gating between detail and context streams, and progressive channel sculpting for feature refinement.

The complete architecture appears in Figure 5, depicting information flow from channel splitting through reciprocal modulation to progressive refinement.

Given input features $F\in R^{B\times C\times H\times W}$, we perform channel-wise splitting into detail and context streams at a ratio β=1/4:

(20) $$F_{\det},F_{\mathrm{ctx}}=\Psi_{\mathrm{split}}\left(F,\left[\beta \cdot C,\left(1-\beta \right)\cdot C\right]\right)$$

where $F_{\det}\in R^{B\times \beta C\times H\times W}$ and $F_{\mathrm{ctx}}\in R^{B\times (1-\beta )C\times H\times W}$.

Detail stream constructs boundary-sensitive texture representations through cascaded convolutions with progressively expanding receptive fields:

(21) $$D=\Phi_{\mathrm{proj}}^{\det}\left(\sigma \left(\Phi_{3\times 3}^{\left(2\right)}\left(\sigma \left(\Phi_{3\times 3}^{\left(1\right)}\left(F_{\det}\right)\right)\right)\right)\right)$$

where $\Phi_{3\times 3}^{\left(i\right)}\left(\cdot \right)$ denotes the *i*-th 3×3 convolution operation, $\Phi_{\mathrm{proj}}^{\det}\left(\cdot \right)$ implements 1×1 projection, and σ(·) denotes ReLU activation.

Context stream preserves high-level semantic abstraction and illumination-related trends with minimal spatial distortion:

(22) $$C=\Phi_{\mathrm{proj}}^{\mathrm{ctx}}\left(F_{\mathrm{ctx}}\right)$$

where $\Phi_{\mathrm{proj}}^{\mathrm{ctx}}\left(\cdot \right)$ denotes 1×1 projection convolution maintaining semantic integrity while adjusting channel dimension.

Reciprocal gating mechanism enables bidirectional information flow between streams. The context stream generates a spatial attention map $A_{s}\in R^{C_{\mathrm{out}}\times H\times W}$ encoding the per-pixel importance derived from global semantic understanding:

(23) $$A_{s}=\sigma \left(\Phi_{\mathrm{BN}}\left(\Phi_{\mathrm{gate}}^{s}\left(C\right)\right)\right)$$

where $\Phi_{\mathrm{gate}}^{s}\left(\cdot \right)$ generates spatial gate through convolution, $\Phi_{\mathrm{BN}}\left(\cdot \right)$ denotes batch normalization, and σ(·) denotes sigmoid activation mapping to [0,1].

The detail stream generates channel attention vector $A_{c}\in R^{C_{\mathrm{out}}\times 1\times 1}$ encoding channel-wise reliability derived from local texture analysis:

(24) $$A_{c}=\sigma \left(\Psi_{\mathrm{GAP}}\left(\Phi_{\mathrm{DW}}\left(D\right)\right)\right)$$

where $\Phi_{\mathrm{DW}}\left(\cdot \right)$ implements depthwise separable convolution capturing local patterns before pooling, $\Psi_{\mathrm{GAP}}\left(\cdot \right)$ denotes global average pooling aggregating spatial statistics, and σ(·) applies sigmoid activation.

These complementary attentions modulate respective streams in reciprocal fashion:

(25) $$D_{\mathrm{mod}}=A_{s}⊙D,\;C_{\mathrm{mod}}=A_{c}⊙C$$

where ⊙ denotes element-wise multiplication with $A_{c}$ appropriately broadcast along spatial dimensions.

Modulated dual-stream features fuse through concatenation, followed by 1×1 projection and residual connection:

(26) $$F^{\left(1\right)}=F+\Phi_{\mathrm{fuse}}\left(\Psi_{\mathrm{concat}}\left(D_{\mathrm{mod}},C_{\mathrm{mod}}\right)\right)$$

where $\Phi_{\mathrm{fuse}}\left(\cdot \right)$ implements fusion convolution and $\Psi_{\mathrm{concat}}\left(\cdot \right)$ concatenates along the channel dimension.

Progressive channel sculpting further refines channel-wise responses to suppress residual non-diagnostic patterns. Intermediate features undergo channel expansion followed by path splitting:

(27) $$\left[X,V\right]=\Psi_{\mathrm{split}}\left(\Phi_{\mathrm{expand}}\left(\Phi_{\mathrm{LN}}\left(F^{\left(1\right)}\right)\right),\left[C_{h},C_{h}\right]\right)$$

where $C_{h}=\left\lfloor 2C/3\right\rfloor$, $\Phi_{\mathrm{LN}}\left(\cdot \right)$ implements layer normalization, $\Phi_{\mathrm{expand}}\left(\cdot \right)$ executes channel expansion convolution, and $\Psi_{\mathrm{split}}\left(\cdot ,\left[\cdot ,\cdot \right]\right)$ splits into the main path *X* and gating path *V*.

The spatial gating unit computes:

(28) $$Z=\Psi_{\mathrm{GELU}}\left(\Phi_{\mathrm{DW}}^{3\times 3}\left(X\right)\right)⊙V$$

where $\Phi_{\mathrm{DW}}^{3\times 3}\left(\cdot \right)$ denotes depthwise 3×3 convolution capturing local spatial context, $\Psi_{\mathrm{GELU}}\left(\cdot \right)$ implements Gaussian Error Linear Unit providing smooth nonlinearity, and ⊙ denotes element-wise multiplicative gating.

The final output integrates projection and residual connection:

(29) $$F_{\mathrm{out}}=F^{\left(1\right)}+\Phi_{\mathrm{out}}\left(\Psi_{\mathrm{dropout}}\left(Z\right)\right)$$

where $\Phi_{\mathrm{out}}\left(\cdot \right)$ implements output projection convolution and $\Psi_{\mathrm{dropout}}\left(\cdot \right)$ applies dropout regularization during training.

## 4. Results

This study evaluates the proposed Endo-DET framework on the EDD2020 benchmark. We present the experimental setup, ablation studies, comparative analysis, and qualitative evaluation. All experiments used identical training configurations.

### 4.1. Datasets and Experimental Setup

#### 4.1.1. Datasets

Dataset 1: EDD2020  [27]: This multi-center endoscopic disease detection benchmark contains 385 video frames from 137 patients across four clinical centers, comprising 749 annotated instances split 8:1:1 into training, validation, and test sets. The dataset covers five disease categories: Barrett’s esophagus (BE), suspicious lesions, high-grade dysplasia (HGD), cancer, and polyps. Multi-modal imaging characteristics span white-light endoscopy, narrow-band imaging, and chromoendoscopy, representing the real clinical challenges of severe class imbalance and lesion morphology diversity.

Dataset 2: Kvasir-SEG [18] This high-quality polyp detection dataset, extracted from the comprehensive Hyper–Kvasir gastrointestinal dataset published in Scientific Data, contains 1000 polyp segmentation images with pixel-level annotations. We converted provided segmentation masks and bounding box annotations for the detection format, splitting the dataset 8:1:1 to evaluate Endo-DET’s performance in single-class polyp detection with high-quality annotations.

Dataset 3: PolypGen2021 [11]: This multi-center polyp detection benchmark published in Scientific Data contains 3057 annotated images from six clinical centers (2445 training, 305 validation, and 307 test) with 3447 gastroenterologist-verified polyp annotations. The dataset encompasses multiple polyp sizes classified as small (≤100×100 pixels), medium (>100×100 to ≤200×200 pixels), and large (≥200×200 pixels), with diverse viewpoints, occlusions, and instrument artifacts.

Dataset 4: CVC-ClinicDB [28]: This widely adopted polyp detection benchmark contains 612 frames extracted from 29 distinct colonoscopy video sequences from 25 patients, each with pixel-level polyp annotation. We converted the segmentation masks to bounding box format and split them 8:1:1.

Data Splitting and Leakage Control: To ensure evaluation integrity, we conducted a post hoc leakage audit on all four datasets using MD5 exact-duplicate detection and 256-bit perceptual hashing (pHash, Hamming distance threshold ≤10) across every train/validation/test boundary.

EDD2020, Kvasir-SEG, and CVC-ClinicDB were each split 8:1:1 at the image level into training, validation, and test sets. None of these datasets provide patient- or examination-level identifiers in their public releases—a limitation shared by the majority of studies employing these benchmarks. Our leakage audit confirmed zero cross-split duplicates or near-duplicates for EDD2020, and found only one and five near-duplicate candidates for Kvasir-SEG and CVC-ClinicDB, respectively, with negligible contamination rates. This limitation is discussed in Section 5.3.

For PolypGen2021, the original image-level random split exhibited 341 cross-split near-duplicate pairs due to temporally adjacent video frames from the same colonoscopy sequences. To eliminate this contamination, we adopted a center-plus-sequence group-level split: centers C1–C4 for training, C5 for validation, and C6 for testing. Under this stricter protocol, the near-duplicate count dropped to zero. Both DEIM-nano and Endo-DET were retrained under identical hyperparameters with this corrected split. The group-level results are reported in Section 4.4 and compared with the original split in Appendix A.2 Table A2.

All four datasets were independently trained and evaluated; no cross-dataset transfer was performed.

#### 4.1.2. Evaluation Metrics

We adopted COCO-style Average Precision metrics for the performance evaluation. AP denotes per-category Average Precision, while mAP (mean Average Precision) denotes the mean across all categories. Throughout this paper, mAP is used for overall multi-category results and AP is used for per-category analysis. mAP50 measures detection accuracy at IoU threshold 0.50. mAP75 provides stricter localization evaluation. mAP50-95 averages across IoU thresholds from 0.50 to 0.95 in 0.05 steps, comprehensively assessing detection quality.

To assess statistical robustness under limited sample sizes and class imbalance, we employed image-level bootstrap resampling on the test set of each dataset. For each evaluation, we performed B=1000 bootstrap iterations with replacement and computed all metrics on each resampled subset. We reported 95% confidence intervals (CIs) using the percentile method with a fixed random seed for reproducibility.

#### 4.1.3. Experimental Platform

All experiments were run on a workstation with NVIDIA RTX 3090 GPU (24GB memory) at batch size 16. Environment configuration: Ubuntu 22.04 LTS, Python 3.10.14, PyTorch 2.2.2 (CUDA 12.1), and TorchVision 0.17.2.

#### 4.1.4. Training Strategy for DEIM and Endo-DET

We designed a two-phase training strategy tailored to endoscopic disease detection characteristics, totaling 400 epochs. We used the AdamW optimizer with momentum coefficients $\beta_{1}=0.9$ and $\beta_{2}=0.999$, with a weight decay factor 0.05. The learning rate scheduling followed theFlat-Cosine strategy: the first 2000 iterations used a quadratic warmup smoothly increasing learning rate from 0 to base rate, followed by 198 epochs of flat phase maintaining a constant rate, then 12 epochs of stable rate, ensuring convergence.

Phase I (epochs 1–198) focuses on foundational feature learning. The first four epochs use only basic augmentation (random horizontal flip and size normalization). Epochs 4–198 apply MixUp augmentation at 0.5 probability, promoting robust feature representation learning.

Phase II (epochs 199–388) initializes from Phase I’s best checkpoint, resets exponential moving average (EMA) decay coefficient to 0.9999, and disables all data augmentation, letting the network focus on learning original data distribution. Epochs 389–400 serve as the final stabilization phase.

### 4.2. Ablation Studies

#### Progressive Validation of Core Components

To validate the effectiveness of proposed domain-specific modules in multi-class endoscopic disease detection, we conducted systematic ablation experiments, progressively integrating ALDF, LUCID, and GLIM-Neck into the DEIM-nano baseline. All configurations were trained for 400 epochs on the EDD2020 dataset using identical training strategies, ensuring fair comparison.

As shown in Table 1, the baseline model (ID 1) achieves 16.4% mAP50-95 and 62.1% Recall50. After adding only ALDF (ID 2), Recall50 improves to 72.6%, a 10.5 percentage point relative improvement attributable to its sparse simplex projection mechanism prioritizing lesion-discriminative tokens over background mucosa through adaptive top-k attention filtering. However, mAP75 drops from 20.1% to 17.3%, indicating a precision–recall trade-off in which improved sensitivity comes at the cost of less precise boundary localization.

LUCID alone (ID 3) pushes mAP50 to 33.0%, achieving the highest coarse localization accuracy among single-component configurations. The mAP75, however, drops sharply to 11.8%, exposing a critical flaw: without illumination normalization, LUCID’s spatial gating mechanism is misled by strong illumination changes, leading to unstable boundary predictions under narrow-band imaging and chromoendoscopy modalities.

GLIM-Neck alone (ID 4) shows the most balanced performance among the single-component configurations, achieving 19.6% mAP50-95 and 23.6% mAP75, improvements of 3.2 and 3.5 points, respectively, over the baseline. Its illumination normalization capability enables stable feature representation across white-light endoscopy, narrow-band imaging, and chromoendoscopy modalities. Notably, GLIM-Neck introduces 711K parameters but only 0.61 GFLOPs, demonstrating a favorable efficiency–accuracy trade-off.

Complete Endo-DET configuration (ID 8) integrates all three core modules, achieving 22.2% mAP50-95, 35.7% mAP50, and 26.2% mAP75, improvements of 35.37%, 39.61%, and 30.35% over the baseline on all COCO metrics, demonstrating optimal performance. More importantly, the recall metrics show larger gains. Recall50-95 improves from 38.9% to 49.8%. Recall50 improves from 62.1% to 75.4%. Recall75 improves from 41.0% to 52.9%.

### 4.3. Comparison with State-of-the-Art Methods

To validate Endo-DET’s effectiveness, we conducted comprehensive comparisons with representative detectors on the EDD2020 dataset. The experiments selected mainstream object detection models including two-stage detectors (Faster R-CNN, Dynamic R-CNN, Cascade R-CNN), single-stage detectors (YOLO series, SSD, Hyper-YOLO, Mamba-YOLO), and Transformer-based detectors (RT-DETR series, DEIM series), covering multiple detection paradigms.

**Figure 6:**
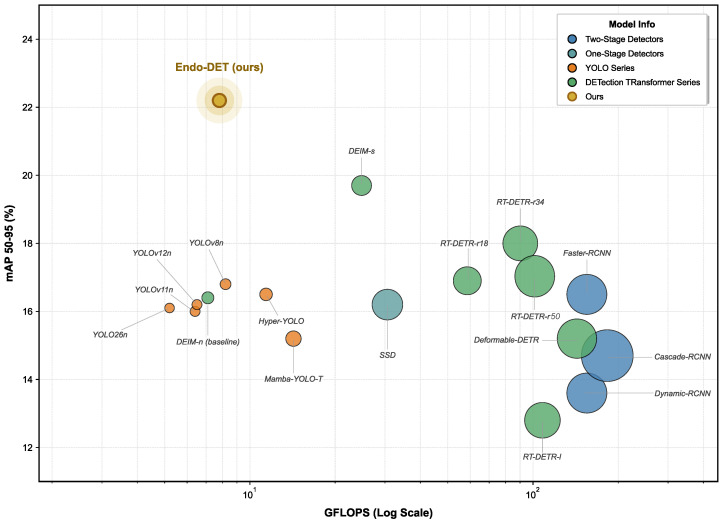
Performance–efficiency trade-off on the EDD2020 dataset. Each bubble represents a detector, with horizontal position indicating computational cost (GFLOPs, log scale), vertical position indicating mAP_50–95_ (%), bubble size reflecting parameter count, and color denoting detector family. Endo-DET (upper-left region) achieves the highest mAP_50–95_ among all methods while maintaining a computational cost comparable to lightweight YOLO variants.

To ensure fair and reproducible comparisons, all methods were evaluated under a unified protocol: identical patient-level train/validation/test splits, the same COCO evaluation toolkit, and best-checkpoint selection on the validation set. Training hyperparameters followed each method family’s officially recommended recipe to ensure optimal performance per architecture. Table 2 summarizes the shared evaluation protocol, and Table 3 details method-specific training configurations grouped by architecture family. Within each family, models differ only in their architecture configuration file; all other hyperparameters remained fixed. The results are displayed in Figure 6.

Table 4 shows that Endo-DET achieved an excellent detection performance while maintaining low computational cost. Compared to RT-DETR-r18, Endo-DET reduced Params by 77.6% and GFLOPs by 86.9%, while mAP50 improved from 25.9% to 35.7%. Among lightweight detectors, Endo-DET outperformed YOLOv8-n by 10.2 points in mAP50 and 6.1 points in mAP75. Compared to the previous best lightweight method, DEIM-Nano, Endo-DET improved mAP50 by 0.5 points and mAP75 by 2.4 points, with Recall50 reaching 75.4%. Two-stage detectors like Faster R-CNN and Cascade R-CNN achieved moderate mAP50 scores but their mAP75 dropped sharply to below 22%, indicating severe localization deficiency. Endo-DET’s mAP75 reached 26.2%, demonstrating superior precision in lesion localization.

**Figure 7:**
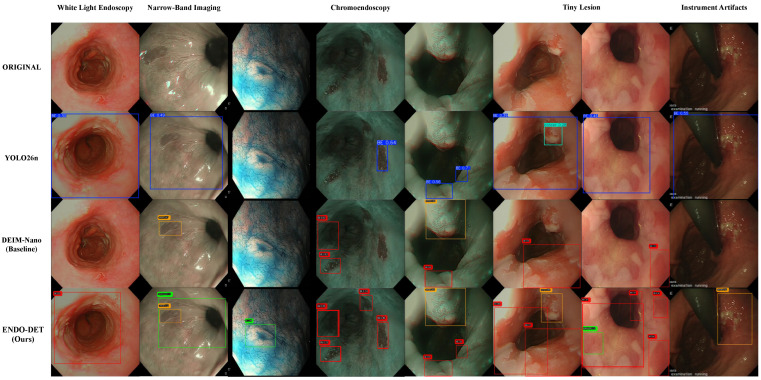
Qualitative detection comparison across five endoscopic scenarios (columns: WLE, NBI ×2, CE ×2, Tiny Lesion, Instrument Artifacts). Rows from top to bottom: original images, YOLO26n, DEIM-Nano (baseline), and Endo-DET (ours). YOLO26n produces oversized bounding boxes in WLE and NBI and misses tiny lesions entirely. DEIM-Nano detects more lesions but with lower confidence. Endo-DET achieves accurate localization with consistently higher confidence scores across all scenarios, including the robust rejection of instrument artifacts (rightmost column).

We further conducted visualization comparisons with mainstream methods (YOLOv8-n and DEIM-Nano) across representative endoscopic scenarios. The visualization experiments selected five challenging scenarios: White-Light Endoscopy, Narrow-Band Imaging, Chromoendoscopy, Tiny Lesions, and Instrument Artifacts, covering diverse imaging modalities and detection difficulties. Figure 7 shows that Endo-DET performed robustly under all scenarios. In the Narrow Band Imaging scenario with characteristic blue-green tint, YOLO26n missed several visible lesions due to color distribution shift, while DEIM-Nano performed better. Endo-DET confidently identified all lesions with accurate bounding boxes. In the Tiny Lesion scenario where lesions occupied less than 0.5% of image area, the YOLO26n completely missed these minute lesions, DEIM-Nano detected some with low confidence, but Endo-DET successfully identified even the smallest lesions. In the Instrument Artifacts scenario, YOLO26n generated false positive on instrument edges and specular reflections, while Endo-DET maintained high specificity by correctly rejecting artifacts. These visualization results demonstrate that Endo-DET’s improvement leads to clinically meaningful detection quality across diverse imaging conditions.

### 4.4. Cross-Dataset Generalization Validation

To comprehensively evaluate proposed domain-specific modules’ generalization capability across different endoscopic scenarios, we conducted validation experiments on three additional polyp detection benchmark datasets. These datasets span different data scales, acquisition centers, and annotation quality, representing diverse clinical application scenarios.

As shown in Table 5, Endo-DET significantly outperforms baseline on all datasets with non-overlapping or minimally overlapping 95% bootstrap confidence intervals, demonstrating statistically robust performance improvement. On Kvasir-SEG dataset, Endo-DET improves mAP50-95 from 46.8% to 57.6%, mAP50 from 72.7% to 80.4%, and mAP75 from 52.3% to 62.6%. On PolypGen2021 dataset with group-level splitting (C1–C4 training, C5 validation, C6 testing) to eliminate near-duplicate leakage, Endo-DET reaches 30.3% mAP50-95, 46.0% mAP50, and 31.9% mAP75, improvements of 4.1, 3.2, and 6.8 points, respectively, over the baseline. The lower absolute performance compared to other datasets reflects the cross-center distribution shift inherent in this stricter evaluation protocol. On CVC-ClinicDB dataset, Endo-DET achieves 68.4% mAP50-95, 91.4% mAP50, and 76.4% mAP75.

### 4.5. Per-Category Detection Performance

To provide a fine-grained understanding of detection capability across disease categories, we report per-category performance on the EDD2020 test set. Table 6 presents category-wise metrics alongside training and test instance counts, enabling direct assessment of the relationship between data availability and detection performance.

Several observations emerge from the per-category results.

Detection performance is strongly associated with both data availability and category-specific morphological characteristics.Among the five categories, HGD achieves the highest AP50 (76.7%) and recall (75.0%), likely benefiting from its relatively distinctive endoscopic presentation with discernible surface texture changes. However, given the limited test set size (seven instances), this metric carries restricted statistical confidence and should be interpreted as indicative of the model’s capacity on morphologically distinctive lesions rather than as a precise performance estimate. BE and polyp, the two most represented categories (220 and 195 training instances), achieve moderate AP50 of 32.4% and 29.3% respectively. Their comparatively lower performance despite larger sample sizes reflects an inherent detection difficulty: BE manifests as subtle mucosal color changes without well-defined boundaries, and polyps exhibit extreme scale variability across the dataset.

Rare categories face fundamental data scarcity constraints. Cancer achieves only 12.3% AP50 with zero recall on the test set, and suspicious lesions reach 27.9% AP50 with 13.0% recall. Both categories show high precision (100.0%) but near-zero recall, indicating that the model adopts an overly conservative detection strategy—it produces very few predictions for these categories, and when it does predict, the predictions tend to be correct. This behavior is a direct consequence of extreme class imbalance during training: with only 46 cancer and 80 suspicious training instances, the model lacks sufficient positive examples to learn robust category-specific representations, and instead learns to suppress predictions for underrepresented categories to minimize overall loss. This data scarcity challenge is not unique to our method; the EDD2020 official benchmark report [10] documented that all participating methods exhibited degraded performance on underrepresented categories, identifying class imbalance as a primary limiting factor for the dataset.

There is an overall precision–recall trade-off. The model achieves 76.0% overall precision but only 30.2% overall recall, reflecting a conservative detection tendency across all categories. This trade-off is partially attributable to the DEIM framework’s one-to-one matching strategy, which inherently produces fewer but higher-quality predictions compared to one-to-many assignment approaches. For clinical deployment, this trade-off can be adjusted through confidence threshold tuning based on specific clinical requirements—lower thresholds would improve recall at the cost of precision, which may be preferable in screening applications, where missing a lesion carries greater clinical risk than a false alarm.

### 4.6. Feature Attention Visualization Analysis

**Figure 8:**
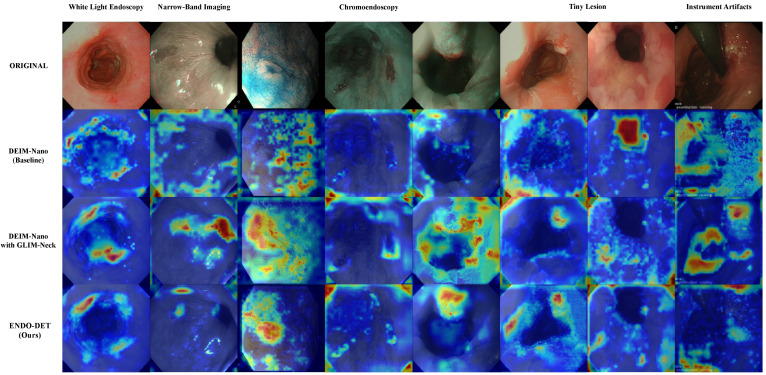
Gradient-weighted Class Activation Mapping (Grad-CAM) visualization across five clinical scenarios (columns: WLE, NBI ×2, CE ×2, Tiny Lesion, Instrument Artifacts). Rows from top to bottom: original images, DEIM-nano baseline, DEIM-nano + GLIM-Neck, and complete Endo-DET. Red/yellow indicates high activation; blue indicates low activation. Progressive improvement is visible across rows: baseline attention disperses over non-diagnostic mucosa, GLIM-Neck concentrates activation toward lesion regions, and complete Endo-DET achieves the most focused activation with clear suppression of instrument artifact responses (rightmost column).

To validate proposed module effectiveness and better understand Endo-DET’s attention mechanism, we conducted a comprehensive visualization analysis through the Gradient-weighted Class Activation Mapping (Grad-CAM) technique, with the results shown in Figure 8. Heatmaps generated through Grad-CAM display attention distribution patterns when different models process typical clinical scenarios, covering five representative challenge scenarios: white-light endoscopy, narrow-band imaging, chromoendoscopy, tiny lesions, and instrument artifacts.

The results show that compared to the baseline, Endo-DET demonstrates significantly more precise and concentrated attention distribution patterns across all test scenarios. In white-light endoscopy scenarios, the baseline activation response disperses, covering large areas of normal mucosa. GLIM-Neck introduction significantly improves activation concentration. Complete Endo-DET further precisely focuses high-response regions on lesion centers, with clear boundaries and obvious response intensity peaks.

### 4.7. Computational Efficiency

Endo-DET achieves a good balance between performance improvement and computational cost. The complete framework contains 4,491,068 parameters and requires 7.84 GFLOPs per image, a moderate overhead compared to the baseline.

Inference speed was benchmarked using TensorRT trtexec (v10.13.3) with FP16 precision on a single NVIDIA RTX 3090 GPU (24 GB, Compute Capability 8.6). The engine was exported with a fixed input shape of 1×3×640×640 (batch size = 1). After a 200 ms warmup phase (39 queries), 995 inference queries were executed over 3.01 s. Table 7 reports the latency breakdown.

The end-to-end host latency, covering host-to-device transfer, GPU computation, and device-to-host transfer, averages 3.25 ms per image, corresponding to a throughput of 330.49 FPS. GPU compute time alone averages 3.02 ms (median 3.01 ms), with P99 latency at 3.31 ms, indicating stable inference with minimal tail latency. Including CPU-side preprocessing (mean 0.85 ms), the total pipeline latency is approximately 4.10 ms per image (∼244 FPS), which substantially exceeds the 25–30 FPS real-time requirement for video-rate endoscopy.

## 5. Discussion

### 5.1. Core Findings

Endo-DET achieves consistent performance improvements on multi-class endoscopic disease detection tasks. On the EDD2020 benchmark, compared with the DEIM-nano baseline, it achieves an improvement in mAP50-95, from 16.4% to 22.2%. Recall metrics show larger gains. Recall50 improves from 62.1% to 75.4%. Recall75 improves from 41.0% to 52.9%. These recall improvements carry critical clinical value in medical screening applications.

Cross-dataset validation confirms method generalization capability. On three independent benchmarks, including Kvasir-SEG, PolypGen2021, and CVC-ClinicDB, Endo-DET achieves mAP50-95 improvements of 10.8, 4.1, and 10.1 points respectively, demonstrating consistent performance improvement across different data distributions, imaging equipment, and annotation quality.

Per-category analysis (Table 6) reveals that detection performance is governed by the interplay between data availability and morphological distinctiveness. HGD achieves the highest per-category metrics (AP50 = 76.7%, AP50-95 = 52.7%), though the limited test set size (7 instances) restricts the statistical confidence of this estimate. BE (AP50 = 32.4%) and polyp (AP50 = 29.3%), despite being the most represented categories, face inherently greater detection difficulty due to their subtle mucosal changes and extreme scale variability, respectively.

For rare categories, cancer (AP50 = 12.3%, Recall = 0.0%, 7 test instances) and suspicious lesions (AP50 = 27.9%, Recall = 13.0%, 10 test instances) exhibit limited absolute performance. This outcome is consistent with the EDD2020 official benchmark findings [10], where all participating methods showed substantially degraded performance on underrepresented categories. We note that these results primarily reflect a data scarcity constraint: with only 46 cancer training instances across diverse morphological presentations, no detection architecture can be expected to learn robust category-specific representations from such limited examples. Addressing this bottleneck requires larger-scale, class-balanced datasets.

On computational efficiency, Endo-DET contains only 4.49M parameters and 7.84 GFLOPs. With TensorRT FP16 optimization, it achieves 330.49 FPS throughput (3.25 ms end-to-end host latency), significantly exceeding the 25-30 FPS real-time requirement for clinical video endoscopy.

### 5.2. Method Advantages

Endo-DET’s performance advantage comes from architectural innovation rather than capacity expansion. Compared to DEIM-s (10.18M parameters, 24.84 GFLOPs, 19.7% mAP50-95), Endo-DET uses 44.1% of parameters and 31.6% of computational overhead to reach 22.2% mAP50-95, a 2.5 point improvement. Compared to similar-scale YOLO series models, Endo-DET achieves consistent 5.7-6.2 point superiority on mAP50-95, with the advantages expanding to 4.8-10.7 points on the stricter mAP75 metric.

Three core modules’ synergy produces performance gains beyond simple addition. ALDF improves Recall50 by 10.5 points through sparse simplex projection with only a 2.2% parameter increase, demonstrating strong parameter efficiency. GLIM-Neck’s four-layer synergistic architecture achieves illumination stabilization across white-light endoscopy, narrow-band imaging, and chromoendoscopy modalities, clearly validated in Grad-CAM visualization. LUCID’s dual-stream reciprocal modulation further suppresses instrument artifacts and specular reflections.

Cascaded gain mechanisms revealed in ablation experiments prove design systematicity. The complete model (22.2% mAP50-95) still improves 1.3 points over best dual-component configuration (20.9%), indicating the challenges addressed by three modules are complementary.

### 5.3. Limitations

Class Imbalance and Rare Category Performance: The EDD2020 dataset exhibits a severe class imbalance, with BE (220 training instances) outnumbering cancer (46 training instances) by approximately 5:1. This imbalance directly constrains detection performance for rare but clinically critical categories. Cancer detection achieves only 12.3% AP50 with zero recall on the test set, and suspicious lesion detection remains limited at 27.9% AP50 with 13.0% recall. While Endo-DET’s architectural innovations improve overall detection quality, they cannot fully compensate for the insufficient training data in tail categories. This limitation is shared across all methods evaluated on EDD2020 [10] and represents a dataset-level constraint rather than a method-level deficiency.

Dataset Scale and Statistical Reliability: The EDD2020 test set comprises only 75 instances across five categories, with as few as 7 instances for HGD and cancer. Per-category metrics at this evaluation scale carry limited statistical confidence, and should be interpreted as indicative of performance trends rather than precise point estimates. Establishing statistically robust per-category benchmarks requires evaluation on substantially larger test sets, which the current publicly available multi-class endoscopic datasets do not provide.

Ongoing Data Collection Effort: To address the data scarcity bottleneck identified in our per-category analysis, we are initiating a multi-center data collection effort targeting large-scale, class-balanced endoscopic disease detection datasets with stratified sampling to ensure the adequate representation of rare categories, including early-stage cancer and suspicious lesions, multi-modal coverage spanning WLE, NBI, and CE, and dual annotations providing both bounding boxes and pixel-level segmentation masks.

Prospective Clinical Validation: All experiments in this study used retrospective, publicly available datasets. Prospective validation in real-time clinical workflows, with diverse patient populations, endoscope models, and operator practices, remains necessary before clinical deployment. The cross-dataset experiments (Table 5) provide preliminary evidence of generalization, but cannot substitute for prospective clinical evaluation.

### 5.4. Future Directions

Based on the current research findings, we identify three promising improvement directions.

Pixel-level segmentation could further improve boundary precision. We are developing a DEIM-based real-time instance segmentation extension, aiming to achieve pixel-level boundary delineation while maintaining real-time inference capability (target <50 ms latency).

Larger-scale multi-class dataset construction represents a key direction for advancing the field. Current public datasets’ limitations in scale, class balance, and multi-modal coverage represent a common challenge facing the entire endoscopic AI field. We are initiating a multi-center data collection project targeting over 10,000 annotated images, using stratified sampling to ensure adequate representation of all classes and lesion scales, providing dual annotations with both bounding boxes and pixel-level segmentation, and covering comprehensive multi-modal imaging.

Clinical validation and deployment optimization will follow technical development. Prospective clinical studies will evaluate Endo-DET performance in real-time operation. Model quantization and neural architecture search will enable edge device adaptation. Interpretability mechanisms including attention visualization and confidence calibration will support clinicians establishing appropriate trust levels.

## 6. Conclusions

We presented Endo-DET, a domain-specific detection framework that systematically addresses three core challenges in multi-class endoscopic disease detection: feature redundancy, illumination inconsistency, and scale variability. Through the synergistic integration of Adaptive Lesion-Discriminative Filtering, Global–Local Illumination Modulation Neck and Lesion-aware Unified Calibration and Illumination-robust Discrimination, Endo-DET reaches 22.2% mAP50-95 on the EDD2020 benchmark, achieving 35.37% relative improvement over the baseline, and demonstrates generalization capability across three independent datasets.

While maintaining the real-time inference capability at 330 FPS, Endo-DET achieves superior performance with significantly fewer parameters and less computational overhead than comparison methods, proving domain-specific architectural design effectiveness. Future work will focus on pixel-level segmentation extension, large-scale multi-class dataset construction, and clinical validation, advancing endoscopic AI from laboratory research toward clinically practical systems.

## Figures and Tables

**Table 1:** Ablation study configurations and quantitative results on EDD2020 dataset. ✓/× indicate the module is enabled/disabled. Best results are shown in bold.

ID	ALDF	LUCID	GLIM-Neck	mAP50-95	mAP50	mAP75	Recall50-95	Recall50	Params	GFLOPs
1	×	×	×	16.4	25.5	20.1	38.9	62.1	3.72	7.12
2	✓	×	×	18.2	30.7	17.3	42.6	72.6	3.81	7.27
3	×	✓	×	16.5	33.0	11.8	40.7	69.3	3.76	7.21
4	×	×	✓	19.6	32.8	23.6	45.8	70.6	4.44	7.73
5	✓	✓	×	18.8	27.6	22.2	46.2	74.0	3.78	7.23
6	✓	×	✓	18.8	28.2	22.6	45.7	**80.9**	4.45	7.75
7	×	✓	✓	20.9	35.2	20.7	43.4	73.7	4.47	7.82
8	✓	✓	✓	**22.2**	**35.7**	**26.2**	**49.8**	75.4	4.49	7.84

**Table 2:** Shared evaluation protocol applied to all compared methods.

Setting	Value
Evaluation toolkit	COCO API (pycocotools)
Primary metric	mAP_50–95_
Checkpoint selection	Best validation mAP
Final evaluation	Held-out test set
Data split	Image-level random (EDD2020, Kvasir-SEG, CVC-ClinicDB); group-level (PolypGen2021)
Hardware	Single NVIDIA RTX 3090 (24 GB)

**Table 3:** Method-specific training configurations grouped by architecture family. Within each family, models differ only in their architecture configuration file.

Setting	YOLO Family	RT-DETR Family	Two-Stage & SSD	DEIM & Endo-DET
	_**(v8n, v11n, v12n, Hyper, Mamba-T, 26n)**_	_**(r18, r34, r50, L)**_	_**(Faster/Dynamic/Cascade R-CNN, SSD)**_	_**(DEIM-n, DEIM-s, Endo-DET)**_
Framework	Ultralytics	Ultralytics	MMDetection	DEIM
Optimizer	SGD	AdamW	SGD	AdamW
LR (init)	default	default	0.02 (SSD: $2\;\times \;10^{-4}$)	$8\;\times \;10^{-4}$
LR (backbone)	–	–	–	$4\;\times \;10^{-4}$
LR schedule	Ultralytics default	Ultralytics default	Step (2×)	Flat-Cosine
Weight decay	default	default	$1\;\times \;10^{-4}$ (SSD: $5\;\times \;10^{-4}$)	0.05
Batch size	4	4	4	16
Input size	640 × 640	640 × 640	1333 × 800 (SSD: 300 × 300)	640 × 640
Epochs	400	400	24 (Cascade: 20)	400
Augmentation	Mosaic (full)	Ultralytics default	RandomFlip (SSD: heavy ^†^)	Staged MixUp ^‡^
AMP	Off	Off	On	Off
Workers	8	4	4	4

^†^ SSD augmentation: Expand → MinIoURandomCrop → RandomFlip → PhotoMetricDistortion; ^‡^ Staged MixUp: basic augmentation (epochs 1–4), MixUp p=0.5 (epochs 4–198), no augmentation (epochs 199–400).

**Table 4:** Performance comparison with mainstream detectors on EDD2020 dataset. The best results are highlighted in bold.

Model	Type	mAP50-95	mAP50	mAP75	Recall50-95	Params(M)	GFLOPs
Faster R-CNN [19]	Two-Stage	16.5	**41.7**	6.0	32.6	41.37	155
Dynamic R-CNN [29]	Two-Stage	13.6	34.2	7.7	27.2	41.37	155
Cascade R-CNN [30]	Two-Stage	14.7	36.1	8.1	30.9	69.21	183
SSD [31]	One-Stage	16.2	40.8	3.5	30.6	24.28	30.6
YOLOv8n [32]	One-Stage	16.8	31.0	20.3	39.9	3.01	8.2
YOLOv11n [32]	One-Stage	16.0	31.3	19.2	42.2	2.59	6.4
YOLOv12n [32]	One-Stage	16.2	32.4	21.4	41.5	2.57	6.5
Hyper-YOLO [33]	One-Stage	16.5	34.2	15.5	38.7	4.09	11.4
Mamba-YOLO-T [34]	One-Stage	15.2	32.2	16.8	45.1	6.13	14.25
YOLO26n [32]	One-Stage	16.1	30.9	19.1	41.6	2.38	5.2
RT-DETR-r18 [25]	DETR	16.9	25.9	19.4	45.9	20.18	58.6
RT-DETR-r34 [25]	DETR	18.0	27.6	20.4	35.7	31.33	90.2
RT-DETR-r50 [25]	DETR	17.1	26.3	19.8	45.2	36.49	98
RT-DETR-L [25]	DETR	12.8	23.5	13.8	39.2	32.82	108
DEIM-n (baseline) [16]	DETR	16.4	25.5	20.1	38.9	3.72	7.12
DEIM-s [16]	DETR	19.7	33.1	21.2	46.6	10.18	24.84
Endo-DET (ours)	DETR	**22.2**	35.7	**26.2**	**49.8**	4.49	7.84

**Table 5:** Cross-dataset generalization performance with 95% bootstrap confidence intervals (B=1000). PolypGen2021 uses group-level split.

Dataset	Model	mAP50-95	mAP50	mAP75	Recall50-95
EDD2020	DEIM-n	16.4 [12.5, 22.1]	25.5 [18.2, 35.1]	20.1 [13.1, 28.7]	38.9 [29.1, 47.5]
	Endo-DET	22.2 [18.7, 28.8]	35.7 [28.4, 47.3]	26.2 [18.1, 35.5]	49.8 [43.9, 55.7]
Kvasir-SEG	DEIM-n	46.8 [39.3, 55.8]	72.7 [62.1, 83.2]	52.3 [42.5, 64.9]	67.5 [61.0, 74.3]
	Endo-DET	57.6 [50.6, 65.0]	80.4 [72.2, 87.6]	62.6 [52.1, 73.1]	79.6 [74.7, 84.5]
PolypGen2021 ^†^	DEIM-n	26.2 [22.9, 30.7]	42.8 [37.6, 48.7]	25.1 [20.2, 30.8]	42.6 [38.6, 46.8]
	Endo-DET	30.3 [26.7, 35.1]	46.0 [40.8, 52.3]	31.9 [26.6, 37.7]	53.7 [49.9, 57.8]
CVC-ClinicDB	DEIM-n	58.3 [52.8, 69.6]	81.9 [70.9, 92.4]	67.1 [58.7, 82.2]	66.4 [60.8, 74.0]
	Endo-DET	68.4 [63.5, 79.2]	91.4 [88.2, 98.7]	76.4 [68.2, 90.6]	77.9 [72.4, 85.9]

^†^ Group-level split (C1–C4 train, C5 val, C6 test). See Appendix A.2 Table A2 for image-level comparison; values in brackets denote 95% CI from B=1000 bootstrap iterations.

**Table 6:** Per-category detection performance of Endo-DET on the EDD2020 test set. Instance counts refer to annotated bounding boxes in training and test splits. Precision and Recall are computed at the default confidence threshold. AP metrics follow the COCO evaluation protocol.

Category	Train	Test	Precision	Recall	AP50	AP75	AP50-95
BE	220	29	66.2	29.4	32.4	17.9	20.2
Polyp	195	22	53.7	33.4	29.3	18.0	17.3
Suspicious	80	10	100.0	13.0	27.9	16.0	14.8
HGD	64	7	59.8	75.0	76.7	75.6	52.7
Cancer	46	7	100.0	0.0	12.3	3.3	5.9
All	605	75	76.0	30.2	35.7	26.2	22.2

**Table 7:** Inference latency breakdown of Endo-DET with TensorRT FP16 on a single NVIDIA RTX 3090 GPU. Input shape: 1×3×640×640. GPU stages measured over 995 queries after 200 ms warmup. Preprocessing measured over 1000 iterations.

Stage	Mean	Median	Min	Max	P95	P99
Preprocessing (CPU)	0.85 ms	0.83 ms	0.66 ms	7.85 ms	1.02 ms	1.13 ms
H2D Transfer	0.22 ms	0.22 ms	0.21 ms	0.42 ms	0.23 ms	0.23 ms
GPU Compute	3.02 ms	3.01 ms	2.99 ms	4.22 ms	3.04 ms	3.31 ms
D2H Transfer	0.01 ms	0.01 ms	0.01 ms	0.20 ms	0.01 ms	0.01 ms
Total Pipeline	4.10 ms	4.07 ms	3.87 ms	12.69 ms	4.30 ms	4.68 ms

GPU inference throughput: 330.49 FPS. Total pipeline throughput: ∼244 FPS; TensorRT v10.13.3, FP16 precision, batch size = 1.

## Data Availability

The data presented in this study are available in EDD2020 at https://edd2020.grand-challenge.org/ accessed on 20 January 2026), Kvasir-SEG (https://datasets.simula.no/hyper-kvasir/ accessed on 20 January 2026), PolypGen2021 (https://doi.org/10.7303/syn26376615; mirror: https://github.com/DebeshJha/PolypGen accessed on 20 January 2026), and CVC-ClinicDB (https://polyp.grand-challenge.org/CVCClinicDB/ accessed on 20 January 2026). The source code will be available on (https://github.com/Anorak2004/Endo-DET accessed on 20 January 2026) upon publication.

## Abbreviations

The following abbreviations are used in this manuscript:

DETR	Detection Transformer
DEIM	DETR with Improved Matching
YOLO	You Only Look Once
ALDF	Adaptive Lesion-Discriminative Filtering
GLIM-Neck	Global–Local Illumination Modulation Neck
LUCID	Lesion-aware Unified Calibration and Illumination-robust Discrimination
GCPA	Global Context Pyramid Aggregator
GCT-Head	Global Context Transformer Head
HACA	Hierarchical Asymmetric Channel Allocation
ALGI	Adaptive Local–Global Injection
DW	Depth-wise
COCO	Common Objects in Context
AP	Average Precision
mAP	Mean Average Precision
IoU	Intersection over Union
FPS	Frames Per Second
FPN	Feature Pyramid Network
GPU	Graphics Processing Unit
BE	Barrett’s Esophagus
HGD	High-Grade Dysplasia
NBI	Narrow-Band Imaging
WLE	White-Light Endoscopy
CE	Chromoendoscopy
EMA	Exponential Moving Average
MAL	Matchability-Aware Loss
O2O	One-to-One
Grad-CAM	Gradient-weighted Class Activation Mapping
BN	Batch Normalization
LN	Layer Normalization
ReLU	Rectified Linear Unit
GELU	Gaussian Error Linear Unit

## Appendix A

### Appendix A.1. Leakage Audit Results

To ensure evaluation integrity, we conducted a post hoc leakage audit on all four datasets using MD5 exact-duplicate detection and 256-bit perceptual hashing (pHash, Hamming distance threshold ≤10) across every train/validation/test boundary. Results are summarized in Table A1.

**Table A1:** Post hoc leakage audit results across all datasets. MD5 detects exact duplicates; pHash (Hamming distance ≤10) detects near-duplicates from temporally adjacent video frames.

Dataset	Split Method	MD5 Duplicates	pHash Near-Dup.	Action Taken
EDD2020	Image-level random (8:1:1)	0	0	None required
PolypGen2021	Image-level random (original)	0	341	Re-split (group-level)
PolypGen2021	Group-level (revised)	0	0	–
Kvasir-SEG	Image-level random	0	1	Disclosed; negligible
CVC-ClinicDB	Image-level random	0	5	Disclosed; negligible

### Appendix A.2. PolypGen2021 Split Comparison

Table A2 compares detection performance between the original image-level random split and the revised group-level split for PolypGen2021. The group-level split (C1–C4 training, C5 validation, C6 testing) eliminates all 341 near-duplicate cross-split pairs identified in the leakage audit (Table A1). The lower absolute performance under group-level splitting reflects cross-center distribution shift, which represents a more realistic evaluation of clinical generalization capability.

**Table A2:** PolypGen2021 performance comparison between image-level random split (original) and group-level split (revised). Group-level split eliminates 341 near-duplicate cross-split pairs but introduces cross-center distribution shift, resulting in lower absolute performance.

Split	Model	mAP50-95	mAP50	mAP75	Recall50-95
Image-level (original)	DEIM-n	61.9	82.0	67.6	75.6
	Endo-DET	67.6	86.6	73.7	80.9
Group-level (revised)	DEIM-n	26.2	42.8	25.1	42.6
	Endo-DET	30.3	46.0	31.9	53.7

### Appendix A.3. Complete Per-Category COCO Metrics

Table A3 reports the complete COCO-style per-category detection metrics on the EDD2020 test set, including scale-specific average precision (AP_s, AP_m, AP_l) for small, medium, and large objects respectively.

**Table A3:** Complete per-category detection metrics of Endo-DET on EDD2020 test set following COCO evaluation protocol. AP_s, AP_m, and AP_l denote average precision for small (<32^2^ pixels), medium ($32^{2}$–$96^{2}$ pixels), and large (>96^2^ pixels) objects respectively. “–” indicates no ground truth instances of that size exist for the category.

Category	Train/Test	AP	AP50	AP75	AP_s	AP_m	AP_l
BE	220/29	0.202	0.324	0.179	0.000	0.016	0.332
Suspicious	80/10	0.148	0.279	0.160	–	0.000	0.159
HGD	64/7	0.527	0.767	0.756	–	–	0.527
Cancer	46/7	0.059	0.123	0.033	–	–	0.060
Polyp	195/22	0.173	0.293	0.180	–	–	0.175
All	605/75	0.222	0.357	0.262	0.000	0.008	0.251
